# Calibration of mass spectrometric peptide mass fingerprint data without specific external or internal calibrants

**DOI:** 10.1186/1471-2105-6-203

**Published:** 2005-08-15

**Authors:** Witold E Wolski, Maciej Lalowski, Peter Jungblut, Knut Reinert

**Affiliations:** 1 Max Planck Institute for Molecular Genetics, Ihnestraße 63–73, D-14195 Berlin, Germany; 2 Institute for Computer Science, Free University Berlin, Takustr. 9, 14195 Berlin, Germany; 3 Max Delbrück Center for Molecular Medicine, Robert-Roessle-Str. 10, D-13125 Berlin-Buch, Germany; 4 Max Planck Institute for Infection Biology, Schumannstr. 21–22, D-10117 Berlin, Germany; 5 School of Mathematics and Statistics, Merz Court, University of Newcastle upon Tyne, NE1 7RU, UK

## Abstract

**Background:**

Peptide Mass Fingerprinting (PMF) is a widely used mass spectrometry (MS) method of analysis of proteins and peptides. It relies on the comparison between experimentally determined and theoretical mass spectra. The PMF process requires calibration, usually performed with external or internal calibrants of known molecular masses.

**Results:**

We have introduced two novel MS calibration methods. The first method utilises the local similarity of peptide maps generated after separation of complex protein samples by two-dimensional gel electrophoresis. It computes a multiple peak-list alignment of the data set using a modified Minimum Spanning Tree (MST) algorithm. The second method exploits the idea that hundreds of MS samples are measured in parallel on one sample support. It improves the calibration coefficients by applying a two-dimensional Thin Plate Splines (TPS) smoothing algorithm. We studied the novel calibration methods utilising data generated by three different MALDI-TOF-MS instruments. We demonstrate that a PMF data set can be calibrated without resorting to external or relying on widely occurring internal calibrants. The methods developed here were implemented in R and are part of the BioConductor package mscalib available from .

**Conclusion:**

The MST calibration algorithm is well suited to calibrate MS spectra of protein samples resulting from two-dimensional gel electrophoretic separation. The TPS based calibration algorithm might be used to correct systematic mass measurement errors observed for large MS sample supports. As compared to other methods, our combined MS spectra calibration strategy increases the peptide/protein identification rate by an additional 5 – 15%.

## Background

Proteomics *inter-alia *focuses on the identification of peptides/proteins in complex biological samples [1]. Before the identification of the complex constituents, several separation steps are required to reduce the sample complexity. The classical separation method is the two-dimensional gel electrophoresis [2-5], followed by excision of the detected spots from the gel, digestion with sequence specific proteases and extraction of the cleaved proteins [6,7]. Mass Spectrometric (MS) analysis [8-13] of the resulting mixture of peptides yields a *peptide mass fingerprint *(PMF): a set of measured molecular masses of the proteolytic peptides derived from the analysed protein [14-16].

PMF commonly requires matrix assisted laser desorption/ionisation (MALDI) time of flight (TOF) instruments, capable of high throughput analysis of complex samples with minimal pre-cleanup, high femtomolar range sensitivity and accuracy of peptide molecular mass determination up to 5 – 10 parts per million (*ppm*) [17-20]. Due to the high ion transmission of the TOF mass analyzer, this technique is more sensitive compared with other MS techniques. In relation to Electrospray ionisation (ESI) MS [21], MALDI-MS is more tolerant to sample contamination resulting from salts and detergents often present in protein samples due to the separation method. MALDI-MS and ESI-MS have become the standard high throughput proteome analysis techniques in many research laboratories.

The experimental peptide mass lists are generated by the analysis of TOF spectra [22]. Ideally, the TOF is proportional to the square root of mass over charge . Thus, in order to transform the spectrum from TOF into *m/z*, two calibration constants *A *and *B *are necessary. These can be derived by measuring the flight times *t *of at least two different ions with known masses and fitting them such that . After the transformation from time into *m/z*, the mono-isotopic peptide signals in the spectrum are identified and their intensity is determined by computational methods [23-26]. The lists of the first mono-isotopic peptide peaks – further called *peak-lists *– are used to identify the protein of interest. In order to assign the PMF to a protein in a sequence database, database search algorithms use the match (within a given measurement accuracy) of theoretical peptide masses computed from protein sequence databases [27] with observed MS masses [15,16].

Usually the scoring schemes model the mass frequencies of the proteins and peptides in the sequence databases [24,28-30]. Other properties to be considered include the different sensitivity of detection for individual peptides, known protein modifications, and/or possible mutations [23,31-33], although generally, all popular search scores depend on the *precise *assignment of experimental to theoretical peptide masses.

### Two novel calibration methods

In a high throughput setting [34,35], where the samples are placed on a moving sample support, the calibration coefficients for transforming the TOF into *m/z *differ depending on sample position. This is due to deviations in plate flatness, sample topography changing the size of the acceleration region [34,36], and alterations in the strength of the electric field on the sample support borders which influences the drift velocity of the ions [22]. Thus, when calibration constants determined from one position on the sample support are used to calibrate TOF spectra acquired on other positions (a procedure known as *external calibration*), the determined *m/z *values have errors of up to 500 ppm.

Calibration is usually performed using external [36-38] or internal calibrants [39,40], which rely on known masses to calibrate the spectra to common co-ordinates. It must be stressed, that in some cases the signal of a reference compounds might be suppressed by the analyte molecules, thus precluding internal calibration. In other cases, the reference signal may partially overlap with an analyte signal, resulting in an erroneous assignment. A third category of calibration methods is based on the peptide mass rule [23,24]. A major advantage of the latter method is that no internal calibrants are required to calibrate the peak-lists. The limitation of this method is it's sensitivity to the presence of non-peptide peaks in the spectra, and that it completely fails if the number of peptide peaks in peak-lists are small [23,24,39]. Therefore, in practice this method usually is used only to pre-calibrate [24] or to support the results of internal calibration [26,39].

We have developed two novel calibration methods for PMF data. Both calibration methods exploit similarities of peak-lists due to closeness in the origin of the analysed samples. The first method combines the computation of dissimilarities [41] between peak-lists with internal calibration. The second method employs spatial statistical methods [42] to model systematic changes of the calibration-model over the MALDI sample support. The major advantage of the presented methods originates from the fact that the MS calibration derives from samples without internal standards or external calibrants positioned on each sample support.

### Evaluating the methods

To demonstrate the accuracy of our methods, we studied one sample set of 380 mass spectra, consisting of a part of the *Arabidopsis thaliana *proteome study [43]. For this purpose, a MALDI MS sample support in pre-structured [35] (384-well) microtitre plate format was used. The measurements were performed using the *Autoflex *MALDI-TOF MS [44] instrument.

To compare the performance of calibration methods described here with those already published [26,39], we used two different data sets. The first set consisted of 1193 spectra deposited on four pre-structured sample supports and measured on a Reflex MALDI-TOF MS [44] instrument (Reflex data set). Spectra were generated via mass spectrometric analysis of the *Rhodopirellula baltica *proteome (unpublished data). The second set was generated in connection with a proteome study of *Mus musculus *and consisted of 1882 spectra deposited on five pre-structured sample supports and measured on an Ultraflex MALDI-TOF MS [44] instrument (Ultraflex data set).

During MS sample preparation of the Ultraflex data set, standard peptides of known masses (human Angiotensin I – 1, 296.6853*Da*, human ACTH (18–39) 2, 465.1989*Da*) were added before the measurement to the MS matrix. This was done because the data sets were optimised for the calibration methods, which required the internal calibrants. We examined if the standard peaks could be observed in *more than *33% of spectra and if so, we removed the peaks matching these masses from the data set. This procedure was applied in order to simulate a data set not optimised for internal calibration.

The *Rhodopirellula *peptide peak-lists were searched against a Pirelulla database [45] with 13, 331 predicted Open Reading Frames (ORFs). The *Mus musculus *samples underwent searches against the *Mus musculus *entries (69, 343 -sequences) of the NCBI non-redundant protein database [46].

## Results and discussion

### Internal calibration using a pre-calibrated list of calibration masses

Internal calibration is a widely used method in mass spectrometry. This method fails however, either if no peaks matching known masses are present or if MS peak assignment is false. A detailed description of the application of internal calibration in a high throughput-MS setting, addressing the two points is given by *e.g. *Chamrad et al. [39], Levander et al. [40] and Samuelson et al. [26]. In order to avoid the lack of MS peaks matching the known calibration masses the authors used a pre-compiled list, *e.g. *trypsin autolysis peaks and unidentified, frequently observed masses [47].

Chamrad et al. [39] initiated the calibration procedure with searches for matching masses using a relatively large search window and iterated it with an increased accuracy. In this scheme, a large search window allows false assignments for calibration masses to occur more frequently. If a false assignment occurs in the first iteration, then the determined calibration constants are false and the entire calibration would be wrong. In the next round of calibration, where a search for matching masses is performed with a higher mass accuracy, the calibration would also fail. To prevent this, the authors [26,39] checked the obtained calibration coefficients against the peptide mass rule (PM-rule) [24,48] and stopped further calibration attempts where they disagreed substantially.

Levander et al. [40] introduced an adaptive method to eliminate low-sensitivity auto-proteolysis trypsin peaks from the calibration mass list if no high-sensitivity trypsin peaks *e.g. *(842.5099*Da*, 1045.5642*Da*, 2211.1046*Da*) were found to decrease the chance of false matches. Unfortunately, this method could only be applied for "tryptic" calibration peaks.

**Figure 1:**
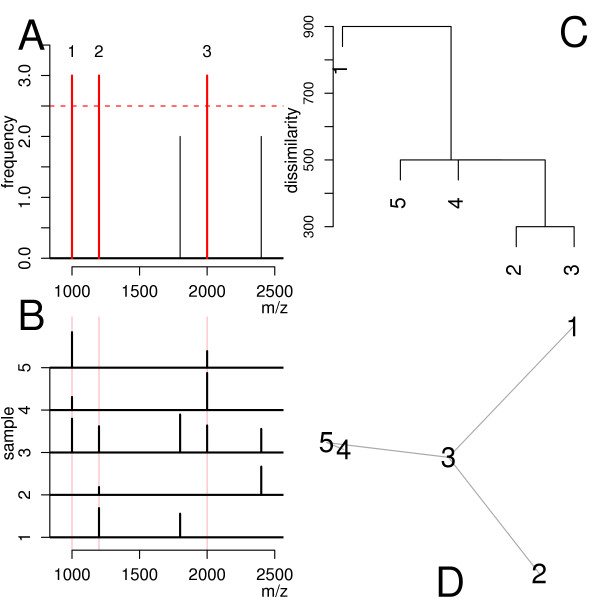
**A**: Histogram of masses present in the stick spectra in **B**. In red, marked masses recognised as ubiquitous. **B**: Stick spectra of five hypothetical peak-lists. Red vertical lines mark the position of ubiquitous masses determined using the histogram in **A**. **C**: Single linkage-clustering dendrogram of the peak-lists in **B**. As dissimilarity the mass measurement range (1500 Da) minus the range enclosed by matching peaks was used. **D**: Minimum spanning tree.

Figures 1A &1B demonstrate the limitations of a calibration list compiled from ubiquitous masses of the whole data set. One can recognise that out of three abundant masses (in red, Figure 1A), only two can be practically used for calibration. Specifically, the first and the third abundant mass in the list of ubiquitous masses (Figure 1A) match simultaneously two peaks in peak-list 3, 4 and 5 (Figure 1B). Thus, out of five peak-lists only three could be calibrated. The second calibration mass is also of no use, since it is the only calibration mass in the peak-lists 1 and 2 (although these peak-lists do contain other shared masses). This illustrates that the usage of a global calibration list may fail to calibrate a set of peak-lists.

It is therefore feasible to address the following questions: How can one obtain a short calibration list to avoid spurious matches while at the same time it matching a sufficient number of peaks in every peak-list of the set? In addition, how can one minimise the initial search window to avoid false matches?

### Finding the optimal multiple peak-list alignment using a modified Minimum Spanning Tree (MST) algorithm

In order to bypass the limitations imposed by global calibration we used an observation made by Schmidt et al. [49]. They noticed that protein samples excised from high-resolution 2D-gels are usually not ideally separated and therefore exhibit local similarities. Compiling a calibration list of abundant masses from a whole data set obtained from a 2D-gel does not differentiate local spectra similarities. For example peak-lists 1, 2 and 3 (Figure 1B) share peaks, which were not recognised as ubiquitous masses and hence not used further for calibration using a global calibration list. The peak-list pairs (2,3) and (1,3) shared more than one peak, thus allowing an easy calibration.

**Figure 8:**
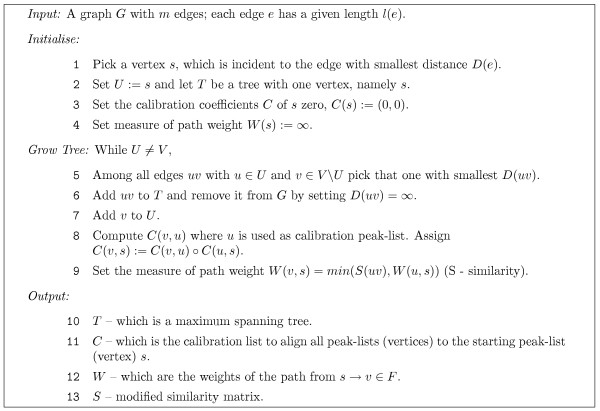
Modified Dijkstra-Prim MST algorithm. The algorithm starts with vertex *s *(peak-list) belonging to the peak-list pair with smallest distance (line 1) (the standard algorithm starts with an arbitrary pair). In addition to computing the MST *T*, the algorithm computes the calibration constants *C*(*v*, *s*) (line 8) and the connection weight *W*(*u*) (line 9).

We explored the property of local pairwise peak-list similarities for calibration of data sets. To achieve it, we used a modified *minimum spanning tree MST *[50] algorithm on the complete weighted graph *G*(*V, E, d*), where the vertex set *V *corresponds to the individual peak-lists and the edges *E *are weighted by a dissimilarity measure *d*. We denned the measure between two peak-lists *p*_1 _and *p*_2 _as *d*(*p*_1_, *p*_2_) = -*s*(*p*_1_, *p*_2_), where *s *represented a similarity measure denned in Equation 10. This measure not only counts the number of matching peaks, but also weights the mass range enclosed by them. Hence, it also considers that if the matching masses lie very close to each other, the calibration model describes a small mass range only, and can result in a large error when aligning masses that are out of this range. Using the dissimilarities one can compute a MST (Figure 1D). The algorithm to compute the MST of the peak-list data set starts by choosing a peak-list (named *s*), which belongs to the peak-list pair of smallest dissimilarity, for example peak-list 2 or 3 in Figure 1. This peak-list is the root of the growing tree *T *(Figure 8 line 1). Next, a peak-list *v *was chosen, which easily could be aligned to peak-list *u *where *v *is a part of the growing tree *i.e. u *∊ *T *(Figure 8 line 5), for example peak-list *v *= 2 can easily be aligned to peak-list *u *= 3. Using linear regression, we computed the coefficients *c*(*v*, *u*) = (*c*_0_, *c*_1_) of the affine function, modelling the absolute mass differences of the peaks matching in the peak-list pair (*v*, *u*). Having these coefficients one can compute the calibration coefficients *c*(*v*, *s*) using the update rule in Equation 11, which described the *mass measurement error *(*MME*) between the peak-list *v *and the starting peak-list s. The calibration is not terminated until the whole tree is built. We then added peak-list *v *to the tree *T *and have iterated the procedure until all peak-lists were appended to the tree, for example by adding peak-list 4, then 5 and finally 1 to *T *(Figure 1D).

In the MST algorithm, the vertices are joined by edges of smallest dissimilarity. Consequently, the MST algorithm connects all peak-lists in the data set in the way that the length of the path from the peak-list of origin (root of the tree: peak-list 3 in Figure 1D) to any peak-list in the data set is minimal. The algorithm for computing the agglomerative clustering using the single linkage method [51,52] works similarly like the MST algorithm and therefore the dendrogram (Figure 1C) provides (as read from bottom to top) the order, by which the peak-list pairs were chosen. The horizontal lines joining two dendrogram tree branches were drawn at the height of the value of the minimal dissimilarity of two peak-lists in either branch.

Finally, the algorithm returns a list of coefficients and a measure of confidence for all peak-lists equalling the smallest similarity in the path from *s *to *v*.

**Figure 2:**
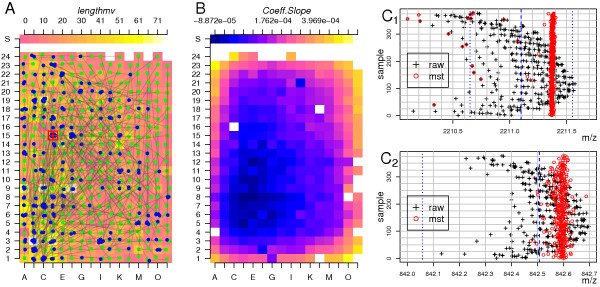
**A**: Colour scheme coded peak-list lengths in dependence of the sample support position. Blue dots – *interior *vertex, Green dots – end vertex, white arrows – connecting edges of the MST. The red hair-cross indicates the peak-list of origin *s*. **B**: Colour scheme coded slope coefficient of the mass- dependent calibration function in relation to sample support position. *C*_1_, *C*_2_: Strip chart of the data set for a mass range of 2210 – 2212*Da *(top) and 842 – 843*Da *(bottom), including the tryptic autolysis peaks 842.508 and 2211.100*Da*. Black hair-crosses – masses of peaks before calibration, red circles – masses after calibration. Vertical blue line – the exact position of trypsin autolysis masses 842.508 and 2211.100*Da*.

Figure 2A demonstrates how the samples on the target are connected by the edges. Green dots (brighter) represent leaves, while blue dots (darker) denote *interior *vertices. The peak-list of origin *s *is marked with a red cross-hairs (sample position D15). Note that long peak-lists (brighter squares) are *interior *vertices of the MST.

The strip-charts of mass ranges including peaks of the trypsin autolysis products 842.508 and 2, 211.100 are presented in Figure 2*C*_1 _and *C*_2_. One can observe that the MST-method works robustly on raw data with a mass measurement error of up to ± 0.7*Da *(black crosses), even if the search for matching peaks when computing the similarities and calibration coefficients was performed within a much smaller window of ± 0.45*Da*. Notably, if the maximal error among two peak-lists is much larger than the search window, it is still possible to find a path, thus allowing alignment of two extreme peak-lists.

Due to the fact that all peak-lists were aligned to the peak-list of origin s, which did not necessarily match to the theoretical trypsin autolysis masses, a final correction was required to calibrate the whole tree to the theoretical co-ordinate system before database searches (not shown).

### Determining the calibration model of the sample support using Thin-Plate Spline interpolation (TPS)

Because a large part of the MME is of systematic origin and depends on the sample support position, the mapping of the calibration coefficients across the entire MALDI plate was introduced by Gobom et al. [36] and Moskovets et al. [38]. The calibration coefficients were determined using a standard mixture of peptides with known masses. Subsequently, the calibration coefficients were used during MS analysis in order to correct for the masses measured afterwards on the same plate.

**Figure 3:**
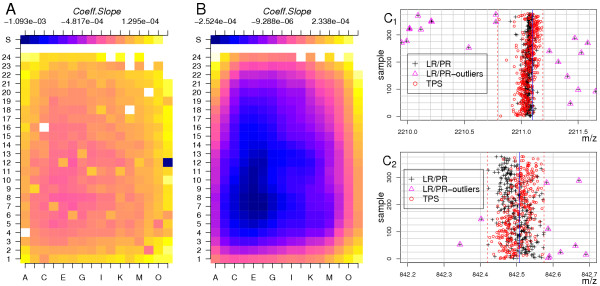
**A**: Colour scheme coded slope coefficients *c*1 of the MME determined by the peptide rule based calibration method. **B**: The slope coefficient as predicted from the refined samples determined by *TPS with λ *= 0.001. **C**: Strip chart of the data set for a mass range of 2210 – 2212*Da *(*C*_1_) and 842 – 843*Da *(*C*_2_), including the tryptic autolysis peaks 842.508 and 2211.100*Da*. Black crosses – masses of peaks predicted by the peptide rule based calibration method, red circles – masses predicted by the TPS calibration method. Vertical blue line – exact position of trypsin autolysis masses 842.508 and 2211.100*Da*. Dashed red vertical line – mass of the extreme peptide masses after TPS calibration.

We introduced here a method that derives the calibration model from calibration coefficients acquired from samples, which do not necessarily contain internal standards. Instead of refining the MST calibration model, we chose the peptide mass rule based approach, namely Linear Regression on Peptide Rule (cf. Methods), to obtain the calibration coefficients. The methods based on the peptide mass rule do not rely on the specification of an initial search window or on internal calibrant masses. The peptide rule based calibration method calibrates the peak-lists into the theoretical co-ordinate system and increases the mass accuracy to approximately 0.1*Da*, but fails if the peak-list is too short, which indeed could be observed for several samples (Figure 3A and 3C). Figure 3A provides the color scheme coded slope coefficient *c*_1 _as determined by the peptide rule based calibration method in dependence of the target location. One can observe that some erroneous predictions occur (Figure 3C; black crosses marked by magenta triangles).

However, it is unbiased to assume a smooth transition between adjacent positions of the sample support. For example, Figure 2B demonstrates that the slope coefficient of the sample calibration-model obtained by the MST calibration methods increases for samples close to the support border. This change is due to alterations in the electric field *E *(Equation 1) influencing the flight velocity given by



where *s*_*a *_is the size of the acceleration region, *z *is the ion charge and *m *is the mass of the ion. We determined the systematic change of the slope using the *Thin-Plate Spline *(*TPS*) interpolation method [42,53]. At first, we computed the TPS with a degree of smoothing *λ *= 5·10^-2 ^(see Equation 15). Calibration models with slope coefficient *c*_1 _that varies more than ± 1·10^-4 ^or with intercept coefficient *c*_0 _varying more than 0.2*Da *from the one predicted by the TPS were discarded. Using the remaining calibration models, the TPS was recomputed with smaller degree of smoothing *λ *= 1·10^-3^. Figure 3B, demonstrates the Colour scheme coded slope coefficient *c*_1_, as estimated by the refined TPS. This model resembles the one generated by the MST method (Figure 2B). We corrected the peak-lists masses (black cross hairs, Figure 3C), using the TPS values as estimates of the slope coefficients, and as intercept estimate we used the average intercept of all coefficients of the refined calibration models to obtain the calibrated masses (red circles).

**Figure 4:**
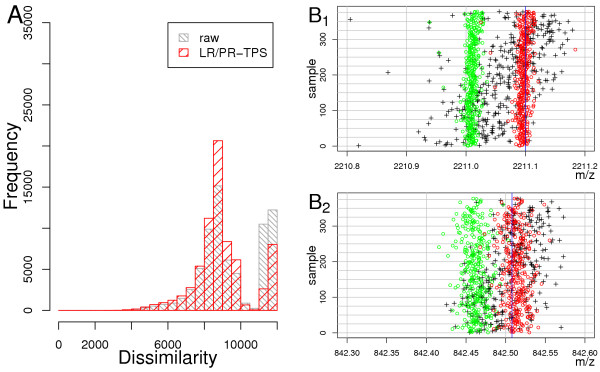
**A**:Histogram of pairwise peak-list similarities. In gray – raw data and similarities computed with an accuracy of ± 0.4*Da*. In red – similarities computed with accuracy of ± 0.15*Da *using LR/PR-TPS calibrated data. **B**: Strip chart of peak-lists. Grey triangles – masses after TPS-calibration, green circles – data after *TPS-MST- *calibration, red circles – data calibrated into the theoretical co-ordinate system, defined by theoretical tryptic autolysis masses (blue vertical lines.)

The TPS method reduced the MME of a peak-list compared to any other peak-list in the data set (vertical red, dashed line in Figure 3C) down to 0.3*Da*, as compared to 1.5*Da *for raw data. This is approximately a 5- fold increase of a mass measurement accuracy. This decrease of the MME enabled us to utilise the MST-algorithm with an accuracy of ± 0.15*Da*, reducing further the probability of false assignments of calibration masses. In addition, the histogram of dissimilarities computed for all peak-list pairs (Figure 4A) shows for TPS calibrated data lower values of dissimilarity (in red) as compared to the raw data (in grey), even if the first dissimilarities were computed with a search window of 0.15*Da *and the second ones with a search window of 0.45*Da*. A subsequent calibration using the MST method decreased further the MME (Figure 4B).

### The mass measurement error

**Figure 5:**
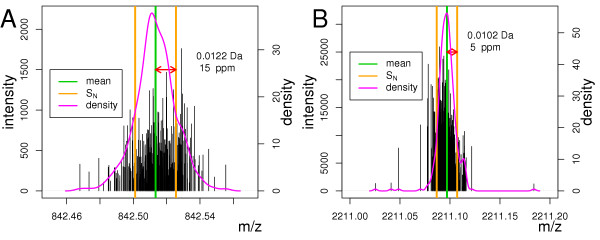
Stick spectrum of the merged data set of 380 peak-lists. The black vertical lines represent peaks calibrated using the TPS and MST method. Their height equals their intensity. Green line – average mass of all peaks in the region 842 – 843*Da *(**A**) and 2210.5 – 2211.6*Da *(**B**). The orange vertical lines represent the average mass ±, the standard deviation of the peak masses in each region. Magenta line – density of peak-masses.

Prior to the calibration, the main error source is due to different drift velocities of the ions causing an increase of the absolute MME, proportional to mass and best described by the slope coefficient *c*_1 _≠ 0 and measured as relative error using parts per million *ppm *(Table 1, row 1 and 2). After removal of this error using calibration methods, for example the TPS calibration (Table 1, row 3,4) or TPS with subsequent MST calibration (Table 1 row 5,6), the main contribution to the MME was due to peak detection performance. We were aware, however, of systematic changes of the MME, which can be described using higher order polynomials [37,54]. We have removed higher order terms of the MME, by applying external calibration before to other calibration procedures (cf. Methods : External Calibration). The change of peak-detection quality was negligible in the range of 500 – 4000*Da*. Figure 5, as well as Table 1, illustrates that after calibration the absolute MME was smaller for the peak with higher mass (2211.1) than that of the peak with a lower mass (842.508) if the peak intensity and consequently the Signal to noise ratio remained sufficiently high. Therefore, we performed the database searches by specifying the search window in *Da *instead of *ppm*.

**Table 1:** Mass Measurement Error. Standard deviation (*S*_*N*_) observed for the trytpic autolysis peaks 842.508 and 2211.1. Raw data; TPS – Thin-Plate Spline (TPS) calibrated data; TPS-MST – The data, which undergone Thin-Plate Spline (TPS)(pre-processing), followed by Maximum Spanning Tree (MST) calibration

Calibration	Mass	*S*_*N*_[*Da*]	*S*_*N *_[*ppm*]
Raw data	842.508	0.1	118
Raw data	2211.1	0.3	135
TPS	842.508	0.03	37
TPS	2211.1	0.057	26
TPS-MST	842.508	0.012	14.5
TPS-MST	2211.1	0.01	4.6

### The optimal size of the search window

Figure 5 and Table 1 demonstrate that it is possible to reduce the mass measurement error to approximately ± 10 *ppm *for most of the peak-lists in a dataset consisting of 380 spectra, by applying the TPS-MST calibration sequence. Nevertheless, in this dataset one can observe peak-lists that do not exhibit such high mass measurement accuracy. Consequently, if the database searches were performed with a search window of 10 *ppm*, these PLs would not be identified.

**Figure 6:**
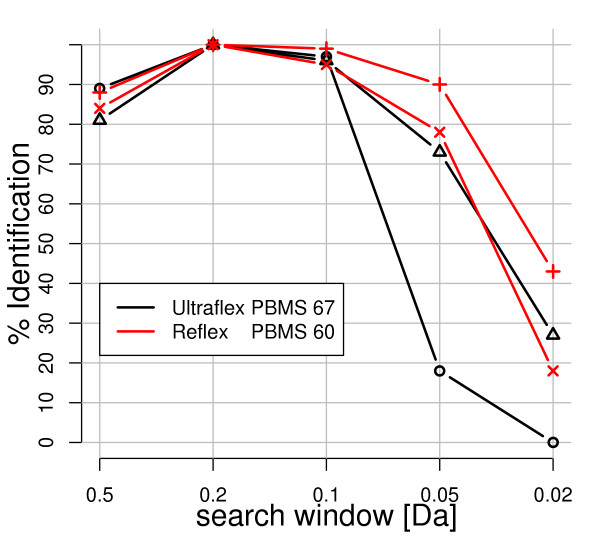
The optimal search window. Comparison of the relative identification rates of internally calibrated data (Y-axis) given a search window size of 0.5*Da*, 0.2*Da*, 0.1*Da*, 0.05*Da *and 0.02*Da*, respectively (X-axis). Red – Two Reflex (Pirellula) dataset, Black – Two Ultraflex (*Mus Musculus*) datasets.

The optimal size of the search window was determined by searching of four *internally *calibrated data sets with five different search window sizes, namely 0.5, 0.2, 0.1, 0.05 and 0.02*Da *using the Mascot [55] search algorithm. The search window of 0.2*Da *generated the highest identification rate. Figure 6 shows the relative identification rate (identification rate / max(identification rate)·100%). Allowing the search window to be larger *e.g. *0.5*Da*, decreases the identification rate by increasing the rate of false negatives, while a smaller window *e.g. *± 0.05*Da *decreases it by rejecting true matches [55]. Because the identification rate for a search window of 0.1*Da *is only slightly worse than one of 0.2*Da*, and since it minimizes the risk of false positive matches, we further compared the practical performance of the calibration methods with a search window of 0.1*Da*.

Prior to the database searches we removed all masses that occur in more than 8% of spectra, as it significantly increased the identification rate [39,40] (cf. Methods – Filtering of ubiquitous masses prior to database search). The sequence data base search was performed using the Mascot [55] search software version 1.8.1. We interfaced the search server from within R using the in-house developed R package msmascot [56].

### Combining different calibration methods and their comparison

All parameters were fitted to a data set optimised for internal calibration, measured on an *Autoflex *MALDI-TOF MS [44] instrument. We applied the calibration methods introduced (MST and TPS based calibration) without changing the parameters to two sample sets obtained using two different instruments, namely a Reflex MALDI-TOF MS and a Ultraflex MALDI-TOF MS instrument. This was executed to illustrate that our methods are robust with respect to different instruments even if the parameters were not optimised for the respective machines.

We combined the different pre-calibration and calibration methods resulting in six different calibration sequences (summarised in Table 2). We compared the performance of the MST and TPS calibration sequence to the internal calibration (IC), and the peptide rule based calibration methods (LR/PR). Furthermore, we investigated if the identification rate of the TPS based method could be improved further by subsequent internal (TPS-IC) or MST calibration (TPS-MST). The R [57] scripts implementing each sequence can be found in the samples directory of the mscalib BioConductor [58] package.

**Table 2:** Calibration sequences. LR/PR – linear regression on peptide rule, IC – Internal calibration with two iterations. (Bruker Reflex – mass measurement error (MME) window of 450 and 250 *ppm*, Bruker Ultraflex 250 and 125 *ppm*); MST – MST calibration method computed with an search window of ± 0.4*Da*; TPS-IC – Pre-processing (TPS calibration) and subsequent internal calibration with a MME window of 250 *ppm*; TPS-MST – pre-processing and an MST with a search window of ± 0.25*Da*;

	Abbreviation	Description
1	LR/PR	peptide rule calibration.
2	IC	internal calibration 450 ppm and 250 ppm.
3	MST	minimum spanning tree calibration.
4	TPS	LR/PR and subsequent thin-plate spline (TPS) calibration.
5	TPS-IC	TPS calibration and subsequent internal calibration.
6	TPS-MST	TPS calibration and subsequent MST calibration.

The only calibration method for which parameters were optimised with respect to the instrument was the standard internal calibration (IC) method, which employs a pre-compiled calibration list of theoretical trypsin autolysis peaks and a calibrated set of ubiquitous masses (cf. Methods – Standard internal calibration). In case of the peptide rule based calibration (LR/PR) method we applied an additional filtering of the calibration-models. Only models with an intercept coefficient *c*_0 _satisfying -0.4*Da *<*c*_0 _< 0.4*Da *and slope coefficients *c*_1 _with -5·10^-3 ^<*c*_1 _< 5·10^-3 ^were kept. In order to avoid falsely calibrated peak-lists we performed the filtering.

The identification rates were defined as the number of identified samples by at least one of the calibration sequences divided by the number of samples submitted for searches



where *CS*_*i *_indicates the set of identified samples by one of the calibration sequences (Table 2), and #{*A*} denotes the number of elements in a set A. The identification rates were 74%, 87%, 79%, 85% for the Pirellula (Reflex) data set, with an overall identification rate of 82%, whereas for the *Mus musculus *(Ultraflex) data set they were 51%, 72%, 35%, 51%, 27%, with an overall identification rate of 58%. The lower identification rate of the *Mus musculus *data set can possibly be explained by the fact that it was matched with a larger database. Therefore, more matching peaks are required to make significant assignments to a data base entry.

**Figure 7:**
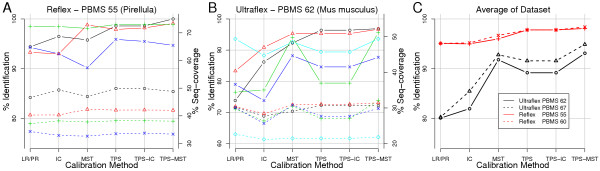
Relative identification rate in % (continuous line – left y-axis) and sequence coverage in % (dashed lines – right y-axis). LR/PR – linear regression on peptide rule, IC – two step internal calibration, MST – minimum spanning tree calibration, P – TPS calibration, TPS-IC – TPS calibration and subsequent internal calibration, TPS-MST – TPS calibration and subsequent MST calibration.

In order to directly compare the identification rates for both data sets and each calibration sequence, we computed the relative identification rate. It was defined as the ratio of the number of identified samples calibrated by a sequence (numerator) and of the number of identified samples, which could be identified by at least one method (denominator):



The relative identification rate is indicated by the dots, joined by continuous lines for readability purposes only, in Figure 7. The dashed lines denote the average of the sequence coverage of all identified samples. Figure 7A presents the results for the four Pirellula data sets, while Figure 7B shows the results of five *Mus musculus *data sets.

Only in one case of one data set was a single calibration sequence TPS-MST (see Table 2) able to identify all peak-lists (100% identification rate) and therefore it completely dominated over the other methods (black line, Figure 7A). In the case of the Ultraflex data set (Figure 7B) we observed that the TPS-MST method had the highest identification rate, while in Reflex data set (Figure 7A) it achieved the highest performance for approximately half of the data sets.

Figure 7C illustrates the averaged relative identification rate of the calibration methods for the Ultraflex and Autoflex data sets. In addition, it demonstrates that the ordering of the calibration methods according to the relative identification rate does not depend on the value of the Probability Based Mowse Score [55] (PBMS) used as identification threshold. The dashed lines (Figure 5) indicate the identification rates obtained for a PBMS 5 units higher than the one used to identify the samples with a 0.5% significance level (continuous lines).

Interestingly, the TPS smoothing method resulted in an overall higher identification rate than the other methods tested on raw data (peptide rule based calibration, internal calibration, MST-calibration), except for one case of the Ultraflex data set. Furthermore, a combination of the internal calibration with TPS calibration (TPS-IC) did not increase either the sequence coverage (dashed lines) or the identification rate of the TPS method applied alone.

In two out of the four Reflex data sets, the MST method applied on TPS-processed data (P-TPS Figure 7A, dashed lines) slightly decreased the sequence coverage indicating a reduction of calibration accuracy. For the Ultraflex data sets, the sequence coverage correlated well with the identification rate and the TPS-MST-method accomplished the highest performance.

Moreover, if similar identification rates of the peptide rule based calibration and the internal calibration were observed, the peptide rule based calibration method provided higher sequence coverage (Figure 7B). This could be explained by the fact that the peptide rule based method calibrated well the peak-lists possessing many peptide peaks. Such peak-lists potentially contain the higher sequence coverage.

### The BioConductor package mscalib

All of the calibration methods are part of the mscalib programme, which is available as a *BioConductor *[59] package. The Bioconductor project is an initiative for the collaborative creation of extensible software for computational biology and bioinformatics [58]. The scripts carrying out the calibration sequences tested, can be found in the subdirectory/*samples *of the package. Furthermore, in the same directory and in the directory/*doc *there are two vignettes [60] with detailed descriptions of two selected calibration sequences.

## Conclusion

While the methods described in this study significantly improve the calibration of raw data, they do not perform better than other published calibration routines which reduce the MME to 10 *ppm *or below. The real advantage of the methods described here is that they are not dependent on the presence of internal or external calibrants, required to correct for the affine component of the MME. Furthermore, the calibration methods described in this study allow a larger fraction of peak-lists in the datasets to be calibrated than the reference internal calibration method would do.

The TPS method deals with systematic detrimental calibration effects that are due to imperfections in the geometry of the electric field over the MALDI sample plates. Usage of TPS calibration results in up to 10% higher identification rates, at least for the Bruker mass spectrometers, than the internal calibration. The TPS calibration procedure enables, for most of the samples deposited on the sample support, to obtain mass accuracy in the range of ± 0.1*Da*. Moreover, the TPS method does not require the presence of internal calibrants since it relies on calibration coefficients acquired from a calibration method based on the peptide mass rule.

The MST method is able to increase the identification rates obtained by the TPS-method for protein samples separated by a 2D-Gel electrophoretic procedure. Furthermore, the parameters optimised for one instrument (Autoflex) can be directly utilised for other instruments (Reflex, Ultraflex).

In this work, we have only examined a version of the MST algorithm that builds a single tree for all peak-lists. This is adequate if the data are a set of peak-lists with smooth transitions in the similarity values. If this is not the case, it might be more appropriate to compute a forest of several MSTs. We have examined, however, only a single peak-list similarity measure (Equation 10) for peak-lists calibration. It is possible that better similarity measures can still be generated and subsequently applied for peak-lists calibration.

Complete utilisation of microtitre plates and sample supports is not only rational with respect to increased accuracy of the TPS method, but also with respect to the idea of high throughput experiments – maximal utilisation of energy and resources. Dense excision of spots from 2D-gels not only increases the performance of the MST method, but also identifies novel proteins. Hence, the main contribution of this manuscript is to present two calibration methods, compatible with the principle of high throughput sample processing and aims to identify a maximum of the proteins resolved on 2D-gels.

However, no single "best-calibration" method exists. Each of the methods utilises different properties of the peak-lists. Consequently, applying these methods in parallel and determining the total (union) of the identified samples provides the highest identification rate.

## Methods

### Data sets

In this study, we used three data sets generated in different proteome analyses:

1. A bacterial proteome *Rhodopirellula baltica *(unpublished data) (1,193 spectra) measured on a Reflex III [44] MALDI-TOF instrument.

2. A mammalian proteome *Mus musclus *(1,882 spectra) measured on Ultraflex [44] MALDI-TOF instrument.

3. A plant proteome *Arabidopsis thaliana *[43] measured on an Autoflex [44] MALDI-TOF instrument.

All PMF MS spectra derive from tryptic protein digests of individually excised protein spots. For this purpose, the whole tissue/cell protein extracts of the former mentioned organisms were separated by two-dimensional (2D) gel electrophoresis [4] and visualised with MS compatible Coomassie brilliant blue G250 [43]. The MALDI-TOF MS analysis was performed using delayed ion extraction and by employing the MALDI AnchorChip ™targets (Bruker Daltonics, Bremen, Germany). Positively charged ions in the range of 700 – 4, 500 *m/z *were recorded. Subsequently, the SNAP algorithm of the XTOF spectrum analysis software (Bruker Daltonics, Bremen, Germany) detected the monoisotopic masses of the measured peptides. The sum of the detected monoisotopic masses constitutes the raw peak-list. Before affine mass calibration, mass measurement errors which can be described by higher order polynomials and determined using external calibration (cf. Methods: External Calibration), were removed. Processed peak-lists were then used for the protein database searches with the Mascot search software (Version 1.8.1) [55], employing a mass accuracy of ± 0.1*Da*. Methionine oxidation was set as a variable and carbamidomethylation of cysteine residues as fixed modification. We allowed only one missed proteolytic cleavage site in the analysis.

### Describing the Mass Measurement Error (MME) and predicting the correct mass

A mass difference can be described either in absolute Δ_*A *_= *m*_*y *_- *m*_*x*_[m/z] or in relative Δ_*R *_= (*m*_*y *_- *m*_*x*_)·10^6^/*m*_*y*_[ppm] units. The masses in two peak-lists *X*, *Y *were compared to each other and we considered two peaks to *match*, in the case of the absolute error if Δ_*A *_<*a*[*m/z*] and in the case of the relative errors if Δ_*R *_<*a*[*ppm*]. If we plotted Δ_*A *_or Δ_*R *_as a function of *m*_theo_, we observed, besides a white noise component *ε *∝ *N*(0, *σ*^2^), a systematic dependence. This dependence was modelled using a function . Given  we corrected the experimental masses using the equations:





depending on whether the relative or absolute error was used, to obtain corrected masses *m*_corr_.

### Affine MME model

In the first approximation, the MME can be described by an affine function , where *m*_*i *_is the mass of the matching peaks. The intercept and slope coefficients of this function can be determined using linear regression.

If only one matching peak was found or the mass range enclosed by the matching masses was small (*e.g. *less than 200*Da*), as a remedy one can fix:

• the intercept at 0, if absolute difference Δ_*A*_[*Da*],

• the slope coefficient at 0, if relative difference Δ_*R*_[*ppm*]

and determine the slope or intercept respectively from the data.

To correct the experimental masses *m*_exp _we used Equation 5 for the absolute differences Δ_*A *_of matching peaks and Equation 4 in case of relative differences Δ_*R*_.

The difference between theoretical and measured masses is called a mass measurement error MME, while the alignment of *m*_exp _on *m*_theo _an *internal calibration *[23,54,61].

### Determining ubiquitous masses and their filtering

To determine the abundant masses we computed two histograms for each data set. The origin in the first histogram  is *x*_0 _= min (*M*) - *h *and of the second histogram  is *x*_0 _= min (*M*) - *h*/2, where *M *are all masses in the data set and the bandwidth *h *equals the measurement accuracy (in *Da*). We divided the range of *M *into bins of *bandwidth h*

*B*_*j *_= [*x*_0 _+ (*j *- 1)*h*, *x*_0 _+ *jh*], with *j *∈ 1,..., *l*,     (6)

where *l *= (max(*M*) - *x*_0_) mod *h*. Formally the histogram of counts *f *is given by [62]



where *n *represented the number of masses in *M*. If a bin had more counts than a given threshold, the average mass  of all peaks in the bin was computed. In the case of two adjacent or overlapping bins *B*_1_, *B*_2 _with a significant number of counts *c*, we first computed a weighted average of the bin midpoints using the number of counts in each bin as weight



where *m*_1 _and *m*_2 _are the bin midpoints. Afterwards, the average mass  of all peaks in the range *m *± *h*/2 was computed. All peaks with mass *m *∈ [ ± *h*/2] were subsequently removed from the data set. Using two overlapping histograms allows the detection of clusters that are scattered over two adjacent bins in one of the histograms. Different ways to determine ubiquitous masses were used and reported by Levender et al. [40] and Kreitler [63].

### Standard internal calibration – Alignment to a pre-compiled list of calibration masses

Instead of using a predefined list of calibration masses, we chose the calibration masses adaptively. The calibration list consisted of ubiquitous masses determined for the data set (cf. Determining ubiquitous masses). Some of the peaks in the list of ubiquitous masses could be assigned to tryptic autolysis products.

These matches were used to calibrate the abundant masses. The peak-lists in the data set were then aligned to the calibrated list of ubiquitous masses.

### Filtering of ubiquitous masses prior to database search

We removed ubiquitous masses that occurred in more than 7.7% of peak-lists [39,40]. Filtering of ubiquitous masses was performed on a calibrated set of peak-lists. As a result, we could use a small bandwidth of *h *= 0.2*Da *(Equation 6) to determine ubiquitous masses. Next, we checked which of them can be assigned with a significant Probability Based Mascot Score (*PBMS*) to a sequence database entry and subsequently removed these masses from the filtering list. Abundant masses assigned to a database entry usually result from proteins multiply detected on a 2D-gel. The multiple identification is due to different localisation of the protein on the 2D-gel caused by: protein modifications (phosphorylation, glycosylation), different splice variants or by partial protein degradation. Finally, we removed all peaks within the range ± 0.1*Da *around the ubiquitous masses.

### Linear regression and peptide mass rule algorithm

Wolski et al. (publication in preparation) defined the distance measure



which computes given *λ*_*DB *_(the average peptide cluster distance for a sequence database *DB *against which the search is performed, *e.g. λ*_*DB *_= 1.000495) the deviation of a peptide mass difference |*m*_*i *_- *m*_*j*_| from the closest monoisotopic mass predicted by the PM-rule [48]. If there was a linear dependence between |*m*_*i *_- *m*_*j*_| and *d*_*λ *_(*m*_*i*_, *m*_*j*_), then it was caused by the slope of the MME. If we computed all differences |*m*_*j *_- *m*_*i*_| and *d*_*λ *_(*m*_*i*_, *m*_*j*_) for peak pairs *m*_*i*_, *m*_*j *_with |*m*_*i*_, *m*_*j*_| < 1400, we could determine the slope coefficient *c*_1 _using linear regression, while fixing the intercept to zero [64]. In order to make the prediction robust against *e.g. *non-peptide peaks, we used a robust linear regression [65]. We removed the slope by multiplying each mass *m*_*i *_in the peak-list by (1 - *c*_1_). Next, we identified the intercept, which was the average of the distance *d*_*λ *_(*m*_*i*_, 0), and corrected for it.

### External calibration

In order to model higher order systematic changes of mass dependent differences Δ of experimental *m*_exp _and reference masses *m*_theo_, the measurements must be evenly distributed over the whole measurement range [37,66]. To model the dependence Δ ∝ *m *we used a cubic smoothing spline function [67,68], given by Δ = *f*(*m*) + *ε*_*i*_, where *f *is a smooth function, and *ε*_*i *_~ *N*(0, *σ*^2^).

In our study, we used an implementation of the smoothing spline function, provided by B.D. Ripley and Martin Mächler (based on Fortran code of T. Hastie and R. Tibshirani) as part of the **R**-*stats *package. Other non-parametric regression methods like local polynomial regression [69] generated similar results for all types of instruments used in this study.

To obtain equidistantly spaced measurements of known masses, *External calibration *was employed. Some sample spots on the sample support are dedicated to calibration only. Calibration samples, of polymer mixtures [36], which yield equidistant peaks were used to precisely estimate the mass-dependent difference function.

### Similarity/quality measures for internal calibration

Peak-lists can be easily aligned if they contain many matching peaks and the masses of these peaks span a wide mass range. The alignment of a peak-list pair (*X*, *Y*) fails if no matching peaks are found. We described these properties mathematically by the following similarity measure:



where *n *represented the number of matches, while *m*_*i *_and *m*_*j *_were the masses of matching peaks. This measure computed the sum of all mass differences of the matching peaks. The power *p *could be used to weight the large differences stronger.

### Alignment of a set of peak-list using a Minimum Spanning Tree

To align a whole data-set to a single peak-list and to align the peak-lists with the highest similarity given by Equation 10, we computed for all peak-lists pairs a distance matrix *D *by casting the similarities into dissimilarities. This distance matrix can be represented by a complete, weighted graph *G*, where the vertices *V *correspond to peak-lists and the edges are weighted with the pairwise dissimilarity. To connect all vertices in the graph *G *with edges *e *of maximal similarity, the *Dijkstra-Prim *algorithm for finding the Minimum Spanning Tree(MST) [50] was implemented. We present here a modified version of this algorithm (see Figure 8). The algorithm was modified with respect to the starting conditions. As a starting-vertex *s *we chose a vertex incident to an edge of smallest distance. In addition to the MST tree *T*, the algorithm returns also a list of calibration coefficients *C*, which align all peak-lists *V *in the data set to the starting vertex (peak-list) *s*, and a list with connection weights *W*.

By traversing the edges in *T*, we reached each vertex in *G*, starting at *s *via edges with the highest possible calibration similarity (smallest distance). This is because we picked *D*(*uv*) with the smallest possible distance (Figure 8, line 5).

To align peak-list *v *to the starting peak-list *s *we needed to determine the coefficients *C*(*v*, *s*) of the difference function  (Equation 5). We could obtain them from the coefficients *C*(*v*, *u*) and *C*(*u*, *s*) of the pairwise difference function  and  by:



where *e.g. * denotes the slope coefficient, and  the intercept of the function .

#### Proof

The masses of the peak-list pairs (*v*, *u*) as well as (*u*, *s*) can be aligned given the *C*(*v*, *u*) and *C*(*u*, *s*) using the equations



Hence,



*C*(*v*, *s*) was computed online using Equation 11 while growing the tree (Figure 8, line 8). Subsequently, the algorithm returned a list *C *of calibration constants, where *C*(*v*, *s*) described the calibration coefficients allowing to transform peak-list *v *into the co-ordinate system of the peak-list of origin *s*.

In order to gain more confidence in the calibration constants in *C*, the MST algorithm was iterated *n *times. For computing the consecutive. *T*_*i*_, *C*_*i*_, *W*_*i*_, *D*_*i *_with *i *= 2,..., *n *we applied the dissimilarity matrix *D*_*i*-1 _and set as a starting vertex *s*_*i*_= *s*_1 _– the vertex incident to the edge of highest similarity in *D*_1_. The returned *T*_*i*_, *C*_*i*_, *W*_*i*_, *D*_*i *_differed since we removed in iteration *i *- 1 each visited edge (Figure 8, line 6).

The calibration constants *C*_*i*_(*v*, *s*) with *i *= 1,.., *n *should ideally be the same. It is known that *C*_*i*_(*v*, *s*) differ due to alignment errors. Therefore, we computed a weighted average of the coefficients of the difference model. As weight of each model *C*_*i*_(*v*, *s*) we utilised the smallest pairwise calibration similarity *W*_*i*_(*v*) (Figure 8, line 9), on the path from *s *to *v*:



We applied the calibration constants in *C*_*w *_to align all peak-lists to the peak-list *s*.

## Abbreviations

• MME – mass measurement error

• MST – minimum spanning tree.

• MS – Mass Spectrometry.

• TOF – Time of Flight.

• MALDI – Matrix Assisted Laser Desorption Ionization.

• mod – modulo operator.

• TPS – Thin plate spline.

## Authors' contributions

ML, KR and PJ gave initial input to the research.

WEW implemented the *BioConductor *package *mscalib*, *msmascot*, carried out the analysis, visualised the results and wrote the manuscript.

ML wrote essential parts of the manuscript

All authors contributed to the final version of the manuscript and approved it.

## Appendix

### Thin-plate spline

The thin-plate spline is the two-dimensional analogue to the cubic spline in one dimension [42,71]. Let *v*_*i *_denote one of the error model coefficients, *e.g. *intercept, at a target location (*x*_*i*_, *y*_*i*_). A thin-plate spline *f*(*x, y*) is a smooth function which interpolates a surface that is fixed at the landmark points *P*_*i *_= (*x*_*i*_, *y*_*i*_) at a specific height *h*_*i *_A thin-plate spline interpolation function can be written as



where *U*(*r*) = *r*^2 ^ln(*r*) is the radial basis function with . This equation is used to predict an unknown *v *for location (*x, y*), and is the unique solution [42,71] which minimises the equation:



This quantity was called the bending energy of the thin-plate spline function. If noise in the determined coefficients *v*_*i *_is detected, one may wish to relax the exact interpolation requirement (Equation 14). This can be accomplished by multiplying equation 14 with a *regularization *parameter *λ*, a positive scalar, and by adding the residual sum of squares, which gives:



Again, as in case of the cubic smoothing spline with the parameter *λ*, the degree of smoothing can be determined. In our study, we utilised an implementation of the TPS [72], according to Doug Nychka [53].
